# Postoperative hip bracing reduces kinesiophobia in patients undergoing hip arthroscopy: a randomized-controlled trial

**DOI:** 10.1007/s00402-024-05437-9

**Published:** 2024-07-09

**Authors:** Maximilian Fischer, Lars Nonnenmacher, Christian Sobau, Alexander Zimmerer

**Affiliations:** 1 Center for Orthopaedics, Trauma Surgery and Rehabilitation Medicine, University Medicine Greifswald, Greifswald, Germany; 2 ARCUS Kliniken, Pforzheim, Germany; 3 Diakonieklinikum Stuttgart, Orthopädische Klinik Paulinenhilfe, Stuttgart, Germany

**Keywords:** Brace, Hip arthroscopy, Young patient, Rehabilitation, Outcome

## Abstract

**Introduction:**

Detailed postoperative rehabilitation protocols after hip arthroscopy for femoroacetabular impingement syndrome (FAIS) are still a matter of debate. Adjunctive hip bracing represents a promising tool to improve early patients’ mobilization. To present, the effect of hip brace therapy on postoperative functional outcomes and specific patient individual psychologic factors remains controversially discussed. Consequently, we aimed to report postoperative outcomes focusing on hip function, pain and kinesiophobia between braced and unbraced patients.

**Materials and methods:**

A prospective, randomized-controlled trial was conducted, including patients undergoing hip arthroscopy for FAIS. After exclusion, a final study cohort of 36 patients in the intervention group (postoperative hip brace) and 36 patients in the control group (no hip brace) were compared for kinesiophobia (Tampa Scale of Kinesiophobia), pain (Visual analog scale) and joint function (International Hip Outcome Tool-12) within the first six postoperative months.

**Results:**

Hip arthroscopy significantly improved all patient-reported outcomes in both groups. Intergroup analysis revealed significantly lower levels of kinesiophobia in braced patients at 6-months follow up (30.7 vs. 34.1, *p* = 0.04) while not negatively affecting pain and joint function. No intra- and postoperative complications occurred within both groups.

**Conclusions:**

This study could demonstrate that bracing after hip arthroscopy can positively influence kinesiophobia, while the brace did not negatively impact postoperative pain and quality of life. Thus, hip bracing could be a viable assistive therapy in the postoperative rehabilitation phase after hip arthroscopy.

## Introduction

Hip arthroscopy is a well-established procedure to treat intra- and extraarticular hip pathologies in a minimally invasive approach [1–3]. The increasing numbers of hip arthroscopies in femoroacetabular impingement syndrome (FAIS)—as a main cause of hip pain in young and active patients—underline its feasibility in this field of hip preserving surgery [4–7].

Besides continuous improvements in arthroscopic technology and technique, increasing attention has been paid to the individual factors that influence postoperative outcomes [8–11]. In this context, psychological distress connected to persisting pain and reduced mobility demonstrated a significant association with postoperative results in hip-preserving interventions [12–15]. For instance, a high level of postoperative pain can lead to excessive fear of physical activity, known as kinesiophobia [16]. Utilizing the Tampa Scale of Kinesiophobia (TSK) to objective this factor of psychological distress, a high level of kinesiophobia has already demonstrated its association with poor postoperative results across various orthopaedic interventions [17–19]. Thus, there is a clear need for evidence-based intraoperative and postoperative care to improve patient outcomes in this context [20].

In postoperative rehabilitation after hip arthroscopy a recent systematic review found, that hip bracing is a commonly used supportive intervention [21]. Benefits of an postoperative brace therapy are connected to the prevention of excessive joint motion and the offloading of the surrounding muscle, resulting in improved joint stability and the protection of the operative repaired tissue [22, 23]. Nevertheless, some surgeons recommend a postoperative brace therapy only in a few specific arthroscopic procedures [24]. Wearing a brace in patients with patellofemoral pain already demonstrated a positive impact on kinesiophobia, while there is paucity in literature regarding brace therapy after hip arthroscopy for FAIS [25].

It becomes clear, that prospective studies are needed to illuminate the effect of hip brace therapy on postoperative functional outcomes and specific patient individual psychologic factors to further improve rehabilitation protocols.

Therefore, the aims of this study were (1) to compare the postoperative kinesiophobia and pain level between an intervention (hip bracing) and a control (no hip bracing) group within the first six months longitudinally and (2) to report the patient-reported outcome using the iHot-12 score in the short-term follow-up. We hypothesized that the postoperative use of a hip brace leads to an improvement in the mentioned scores.

## Patients and methods

### Study design

**Fig. 1:**
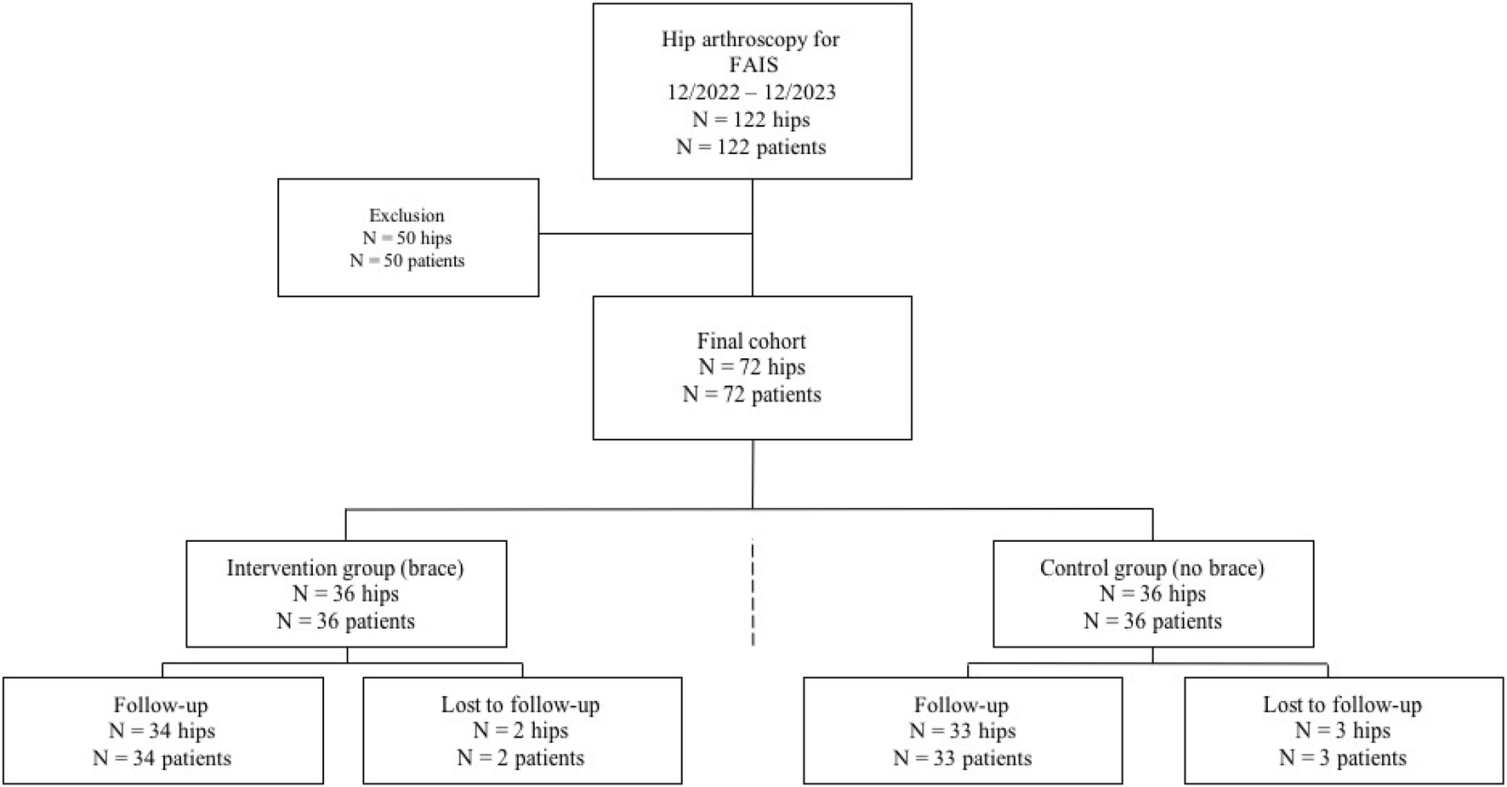
Flowchart of included patients. FAIS, Femoroacetabular impingement syndrome

Inclusion criteria consisted of clinical and radiological diagnosis of symptomatic FAIS, failure of conservative treatment, patient age > 18 years, and the capacity to provide informed permission. Hip dysplasia (lateral center–edge angle of Wiberg (LCEA) < 25°), osteoarthritis (Tönnis grade > 1), history of pediatric hip disorders, chronic pain syndrome, revision hip arthroscopy, and refusal to participate in this study were exclusion criteria. During the selected time period, 122 individuals underwent hip arthroscopy between December 2022 and December 2023. 50 patients were excluded from the analysis due to the aforementioned exclusion criteria, leaving 72 patients available for the study (Fig. 1).

The patients were randomly assigned into an intervention group (postoperative hip brace) and a control group (postoperative routine care without hip brace) resulting in a final study cohort of 36 patients in the study group and 36 patients in the control group. Five patients were lost to follow-up (intervention group: two, control group: three). All included patients completed a frequent clinical follow-up six weeks, three months and six months postoperatively.

### Surgical technique and postoperative regime

Two fellowship-trained hip surgeons (A.Z., C.S) performed all procedures in the supine decubitus position using two to three standardized portals. A post-traction system was used for arthroscopy. During surgery the central and the periphery compartments were accessible. Acetabuloplasty, femoroplasty, and a combination procedure were used to treat pincer, cam, and mixed-type FAIS. If preservation of the labrum was feasible, a labrum repair was performed; otherwise, the labrum was reconstructed. Periportal capsulotomies were performed on each patient. An interportal capsulotomy was performed and later restored in the case of a significant cam morphology.

Postoperatively, a partial weight-bearing of 20 kg for 6 weeks was recommended. Hip flexion was limited to a maximum of 90° for 6 weeks. The intervention group was treated postoperatively by wearing an additional hip brace (HipoCross, Orthoservice AG, Switzerland) for six weeks, whereas the postoperative mobilization in the control group did not include wearing such a hip brace. Continuous passive motion was recommended for four hours daily to reduce intra-articular adhesion risk. A three-week course of oral nonsteroidal anti-inflammatory drugs achieved pharmaceutical prophylaxis of ossification.

### Patient-reported outcomes and statistics

Preoperatively, 6 weeks, three months, and six months postoperatively, kinesiophobia was assessed using the Tampa Scale of Kinesiophobia (TSK), and the pain level was reported using a visual analog scale (VAS). Higher values indicated a higher level of kinesiophobia and pain.

The patients' quality of life was assessed using the International Hip Outcome Tool-12 (iHot-12) preoperatively and six months after hip arthroscopy.

The sample size was calculated to have a power of 0.80 (1-β). Thirty-two patients were required in each group. In consideration of a 10% loss, 36 patients were selected for each group, for a total of 72 patients. G*Power software was used to calculate the sample size (version 3.1.9.4).

Descriptive statistics were used to summarize the patient characteristics and outcomes. Patient-reported outcome data were reported as mean with standard deviation. Xlstat was utilized for statistical calculations (ADDINSOFT, Paris, France). Chi-Square and Fisher Exact tests were employed to compare categorical data. Continuous data were compared using a two-tailed t-test (assuming normally distributed data) and Wilcoxon rank-sum test (for non-normally distributed data). A p-value less than 0.05 was considered statistically significant.

### Ethical considerations

All patients gave written informed consent prior to inclusion. Ethics approval (F-2022-112) was obtained from the local independent ethics committee according to the World Medical Association Declaration of Helsinki. The study was registered within the Federal Clinical Trials Registry (DRKS00030873).

## Results

### Patient demographics

The preoperative patient demographics are presented in Table 1. The mean age at the time of surgery was 37.5 years. 61% of patients were male; the mean body mass index (BMI) was 25.5 kg/m^2^. The intervention group included 59% male patients with a mean age of 36.6 years and a mean BMI of 23.5 kg/m^2^. Compared to this group, the control group included a higher percentage of male patients (78% vs. 59%) and showed a higher mean age (38.5 vs. 36.6 years) and mean BMI (25.6 vs. 23.5 kg/m^2^) at the time of hip arthroscopy.

**Table 1:** Patient demographics

	Total (n = 67)	Intervention (n = 34)	Control (n = 33)
Age (Min – Max)	37.5 (18–65)	36.6 (22–62)	38.5 (18–65)
Sex (% male)	61	59	78
Height m (Min—Max)	1.76 (1.53–1.98)	1.76 (1.53–1.97)	1.77 (1.64–1.98)
Weight kg (Min—Max)	76.8 (50–118)	73.1 (50–97)	80.6 (56–118)
BMI kg/m^2^ (Min—Max)	25.5 (18.3–37.2)	23.5 (18.3–29.2)	25.6 (19.6–37.2)
ASA score, n (%)			
1	49 (73)	24 (71)	25 (76)
2	18 (23)	10 (29)	8 (24)
Procedures, n (%)			
Femoroplasty	59 (88)	29 (85)	30 (88)
Acetabuloplasty	49 (73)	25 (74)	24 (71)
Labral Debridement	10 (15)	5 (15)	5 (15)
Labral Repair	57 (85)	29 (85)	28 (82)
Chondroplasty	15 (22)	7 (21)	8 (24)

Values are shown as n (%) or means ± Standard Deviation (range)

*BMI* Body Mass Index, *ASA* American Society of Anesthesiologists

### Tampa scale of kinesiophobia and visual analog scale for pain

Preoperatively, the mean TSK score showed no significant differences between the intervention and the control group (40.9 vs. 41.6, p = 0.86). Postoperatively, the TSK score declined continuously in both groups compared to the preoperative state (Table 2). The postoperative values were lower in the intervention group compared to controls at all postoperative follow-up time points, without reaching statistical significance six weeks (mean, 37.9 vs. 39.6, p = 0.38) and three months (mean, 34.8 vs. 36.7, p = 0.42) postoperatively.

**Table 2:** Tampa Scale of Kinesiophobia (TSK)

	Intervention (n = 34)	Control (n = 33)	p
Preoperative (mean ± SD)	40.9 (± 5.3)	41.6 (± 6.8)	0.86
6 weeks (mean ± SD)	37.9 (± 6.1)	39.6 (± 6.5)	0.38
3 months (mean ± SD)	34.8 (± 5.9)	36.7 (± 6.2)	0.42
6 months (mean ± SD)	30.7 (± 4.5*	34.1 (± 5.8)*	0.04*

Values are shown as means ± Standard Deviation (SD)

^*^A p-value less than 0.05 was considered statistically significant

At the latest follow-up, the mean TSK score was significantly lower in the intervention group (mean, 30.7 vs. 34.1, p = 0.04).

The preoperative pain level showed comparable values between both groups (mean, 6.4 vs. 6.1, p = 0.86). Hip arthroscopy led to a significant decline of pain in both groups with continuously decreasing values across the postoperative follow-up examinations. Comparing the postoperative pain levels between both groups, there were no significant differences (Table 3).

**Table 3:** Visual analog scale for pain (VAS)

	Intervention (n = 34)	Control (n = 33)	p
Preoperative (mean ± SD)	6.4 (± 2.1)	6.1 (± 2.1)	0.86
6 weeks (mean ± SD)	4.9 (± 2.0)	5.8 (± 2.1)	0.23
3 months (mean ± SD)	3.8 (± 1.9)	3.8 (± 1.9)	0.97
6 months (mean ± SD)	2.4 (± 2.3)	2.5 (± 2.2)	0.87

Values are shown as means ± Standard Deviation (SD)

^*^A p-value less than 0.05 was considered statistically significant

### International hip outcome tool-12

Before hip arthroscopy, there were no significant differences comparing the mean iHot-12 scores between the intervention and the control group (41.9 vs. 42.9, p = 0.83). At the latest follow-up the iHot-12 had significantly improved within both groups (p < 0.001). Intergroup analysis showed no significant difference between the mean scores 6 months postoperatively (61.5 vs. 62.7, p = 0.49) (Table 4).

**Table 4:** International hip outcome tool-12 (iHot-12)

	Intervention (n = 34)	Control (n = 33)	p
Preoperative (mean ± SD)	41.9 (± 15.4)	42.9 (± 16.5)	0.83
Six months (mean ± SD)	61.5 (± 6.4)	62.7 (± 7.4)	0.49

Values are shown as means ± Standard Deviation (SD)

^*^A p-value less than 0.05 was considered statistically significant

None of the patients experienced any postoperative complications, nor were any complications reported as a result of the brace. No patient was taking pain medication at the six-month follow-up.

## Discussion

The main result of the study was, that wearing a hip brace after arthroscopy positively influenced kinesiophobia, while the brace did not negatively impact postoperative pain and quality of life. Thus, hip bracing could be a viable adjunctive therapy to improve patients´ mobility after hip arthroscopy.

Femoroacetabular impingement syndrome (FAIS) is a frequent cause of hip pain in young patients [7, 26]. Detailed rehabilitation protocols after hip arthroscopy for FAIS are not well established yet, even when hip braces are often utilized to assist mobilization after surgery [21, 27]. Based on the idea of tissue protection and muscle offloading, using a hip brace should normalize gait patterns while walking and reducing pain to improve early mobilization [22, 23, 28]. A recent study investigated the biomechanical effects of hip braces on patients after hip arthroscopic surgery for FAIS, finding that wearing a hip brace significantly reduced the peak hip flexion angle and the peak acceleration of the greater trochanter during standing-up and walking tasks at three weeks postoperatively. The authors suggested that hip braces may offer protective benefits for the repaired tissues during the early stages of postoperative recovery for patients undergoing arthroscopic FAI correction and labral preservation surgery [22].

In knee surgery, it has already been demonstrated beneficial impacts on postoperative outcomes. For instance, in patients with high levels of kinesiophobia, a functional brace therapy improved the functional outcome and kinesiophobia postoperatively [16, 29]. After hip arthroscopy, rehabilitation protocols utilizing a brace have poorly been studied and demonstrated conflicting results. Recently, Wentzel et al. studied 193 patients after hip arthroscopy for FAIS and found no significant differences between braced and unbraced patients regarding patient-reported outcomes and reoperation rates [30]. These results are in line with our findings. Nonetheless, factors like mental health or kinesophobia were not considered in the methods. A recently published study by Nasir et al. was able to demonstrate that poor physical function and high pain scores eight weeks after hip arthroscopy were associated with increased kinesiophobia [31]. We could demonstrate, that kinesiophobia was significantly reduced six months after hip arthroscopy in braced patients. A prospective cohort-study by Clapp et al. demonstrated a significant decline in kinesiophobia one year after hip arthroscopy for FAIS [32]. This finding is emphasized by our study, suggesting that a brace seems to enhance the improvement of kinesophobia. Consequently, using a brace after surgery could lead to an improvement in TSK scores when elevated TSK levels are observed before the operation.

Nevertheless, prospective, randomized-controlled trials are needed to enhance the level of evidence for detailed recommendations on postoperative rehabilitation [33, 34]. To date, there is one randomized-controlled trial being conducted (Clinical Trial NCT04599296) on postoperative hip bracing after arthroplasty, but its results are still pending. Therefore, our study represents the first randomized-controlled trial in this area reporting on patient-reported outcome as well as patient individual psychological factors.

While the present study reports beneficial effects of postoperative hip bracing in a prospective, monocenter series, several limitations of this study must be considered. The study included only patients receiving hip arthroscopy to treat FAIS and there could be an increased risk for selection and treatment bias caused by two surgeons in one high-volume center performing all surgeries in this study. Nevertheless, the prospective, randomized study design including a control group should be noted as a strength of this study. However, the study groups were not balanced by sex and the higher rate of male patients in the control group could have influenced the results. Even when sex-related differences in postoperative outcomes have already been reported for different orthopaedic procedures, randomized-controlled trials cannot guarantee sex-balanced study groups. Additionally, multi-center studies could be beneficial to improve the generalizability of the results. Last, the data were limited to a short-term follow-up period of six months. Thus, mid- and long-term outcomes have to be reported in future studies.

Besides all mentioned limitations, the present study demonstrates significant clinical improvement in postoperative patients´ mobilization utilizing a hip brace in a prospective, randomized-controlled trial. The results of this study enhance the understanding of postoperative patient´s care after hip arthroscopy for FAIS substantially. Further research is warranted to improve postoperative rehabilitation recommendations, particularly across variable indications in hip preservation by arthroscopy.

## Conclusion

This study could demonstrate that bracing after hip arthroscopy can positively influence kinesiophobia, while the brace did not negatively impact postoperative pain and quality of life. Thus, hip bracing could be a viable assistive therapy in the postoperative rehabilitation phase after hip arthroscopy.
